# Climatic Determinants of Physicochemical Traits and Probiotic Composition in Dwarf Honeybee ( *Apis florea* ) Honey

**DOI:** 10.1002/fsn3.70640

**Published:** 2025-07-16

**Authors:** Shabnam Parichehreh, Gholamhosein Tahmasbi, Maryam Asnaashari, Fatemeh Ahmadi, Mohammad Eslampanah

**Affiliations:** ^1^ Department of Honeybee Animal Science Research Institute of Iran, Agricultural Research Education and Extension Organization (AREEO) Karaj Iran; ^2^ Department of Animal Processing Animal Science Research Institute of Iran (ASRI), Agricultural Research, Education and Extension Organization (AREEO) Karaj Iran; ^3^ Department of Entomology, Faculty of Agriculture Tarbiat Modares University Tehran Iran; ^4^ Department of Animal Pathology and Epidemiology Razi Vaccine and Serum Research Institute, Agricultural Research, Education and Extension Organization (AREEO) Karaj Iran

**Keywords:** 16S rRNA sequencing, *Apis florea*, environmental influence, honey microbiota, lactic acid bacteria (LAB), probiotics

## Abstract

The presence of lactobacilli in honey is highly significant, primarily due to their potential to inhibit pathogens and confer antimicrobial properties. To investigate this, honey samples were collected from 40 *Apis florea* colonies situated across the southern, southeastern, and southwestern provinces of Iran. Initial screening involved Gram staining and catalase activity analysis of the isolated bacteria. Following genomic DNA extraction from these bacterial colonies, the 16S rRNA genes were amplified via polymerase chain reaction (PCR using LAB‐specific primers (27F and 1492R)). This study successfully employed a targeted methodology to isolate 100 lactic acid bacteria (LAB) strains from *A. florea* honey. Subsequent phylogenetic analysis and phenotypic characterization led to the identification of 19 representative strains closely affiliated with three distinct species: *Lactobacillus kunkeei, L*. *plantarum* , and *Enterococcus faecalis* . Furthermore, the research highlighted the significant influence of climatic factors, such as temperature, precipitation, and sunlight exposure, on key biochemical indicators in *A. florea* honey. These factors markedly affected the levels of sucrose, proline, diastase activity, and hydroxymethylfurfural (HMF), underscoring the critical role of environmental conditions in shaping the honey's composition. Ultimately, the identification and isolation of probiotic strains from honey offer promising avenues for their application in functional foods and nutraceuticals. These potential probiotic candidates could contribute to advancement in gut health, food preservation, and the development of novel bioactive ingredients with diverse therapeutic applications.

## Graphical abstract

This study investigates the relationship between climatic variables and honey's physicochemical and microbiological properties from various regions. It highlights how environmental factors, such as temperature and humidity, influence honey quality, composition, and microbial communities. The findings provide insights into regional differences and contribute to quality assessment and honey authentication.

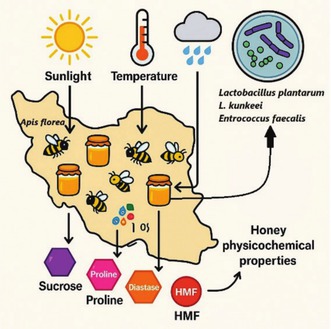

## 1 Introduction

Honey has long been recognized as a nutritious and natural remedy. Bees produce honey from floral nectar, a dense, energy‐rich substance with desirable sensory and health benefits. Its chemical composition and sensory characteristics are primarily influenced by factors like bee foraging behavior, nectar source, and regional environmental conditions. The diverse plant sources contribute significantly to variation in honey's flavor, aroma, and color (Vit et al. 2015).

Honey contains many sugars, with fructose and glucose predominant, along with organic acids, proteins, antioxidants, flavonoids, enzymes, amino acids, and phenolic compounds (Almasaudi 2021; Oryan et al. 2018). Honey's well‐documented health benefits, such as wound healing and antioxidant effects, stem from its complex biochemical composition Erejuwa et al. 2012. Key constituents—including sucrose, diastase, proline, and hydroxymethylfurfural (HMF)—offer important insights into honey's quality, authenticity, and health impact (Gheldof et al. 2002). Sucrose and other sugars affect the glycemic index, which is relevant for individuals monitoring blood sugar levels (Palma‐Morales et al. 2023). Diastase activity indicates honey freshness and proper handling; higher levels reflect minimal heat treatment and better enzyme preservation. Proline, the main amino acid in honey, is widely used to assess maturity and authenticity, as its concentration may reveal adulteration (da Silva et al. 2016). HMF levels, which increase with heat and storage time, can signal overheating or prolonged storage, potentially reducing nutritional value and sensory quality (Yiğit et al. 2024). Assessing these parameters is therefore crucial for evaluating honey's quality and understanding its health benefits.

Various microorganisms are present in honey in low quantities, including fungi, yeast, *Bacillus* species, *Clostridium* species, and lactic acid bacteria (LAB) (Neveling et al. 2012; Vit et al. 2015). LAB, in particular, derives its sustenance from nectar and pollen and interacts with mature honeybees (Endo et al. 2009; Neveling et al. 2012). These microorganisms are especially prevalent in carbohydrate‐rich environments. Several studies suggest that LAB play a significant role in the fermentation process that converts nectar into honey, and transform pollen into bee bread, owing to their fermentative abilities (Vásquez et al. 2009; Olofsson et al. 2016). The microorganisms found in honey originate from both primary and secondary sources, such as the honeybee digestive system and secondary sources, including nectar, pollen, propolis, floral matter, and the hive's environment (Olofsson and Vásquez 2008).

Olofsson and Vásquez (2008) detected the presence of *Lactobacillus*, *Enterococcus*, and *Bifidobacterium* in both the honey stomach and fresh honey of *Apis mellifera* . Similarly, Tajabadi et al. (2011) reported these bacterial genera in the honey stomach and honeycomb of *Apis dorsata* . Hasali et al. (2015) isolated five strains of LAB from Meliponini honey collected from various regions along the East Coast of Peninsular Malaysia. Pajor et al. (2018), examining honey samples from Poland, found that 80.4% of the bacterial isolates belonged to the genus *Bacillus*, with 37 out of 46 strains identified as such. Feizabadi et al. (2021) surveyed stored honey from *Apis mellifera* and successfully isolated both *Enterococcus* and *Lactobacillus* species. In studies by Parichehreh et al. (2017, 2018), multiple strains of *Lactobacillus kunkeii*, *L. plantarum* , *L. apis*, *Enterococcus faecium* , *E. faecalis* , and *E. hirae* were identified from the stomachs of the Asian dwarf honey bee, *Apis florea* , in Iran.

*Apis florea* , commonly known as the Asian dwarf honey bee, is a wild bee species distributed across a broad expanse of Asia. Its range extends approximately 7000 km, from Vietnam and southeastern China through to the southern Himalayas and the Iranian Plateau to southern Oman (Hepburn et al. 2005). This broad geographical distribution likely contributes to considerable variation in the gut microbiota of *A. florea* , which may, in turn, influence honey production. Research by Olofsson and Vásquez (2008) revealed that the composition of LAB in honey varies notably depending on both the floral sources and the season. Recent studies by Parichehreh et al. (2018) successfully isolated and identified several species, such as *Lactobacillus* and *Enterococcus*, from the stomachs of *A. florea* , further highlighting the microbial diversity present within this species.

The diverse climatic conditions across Iran play a significant role in the production of a wide range of honeys, each with sensory, organoleptic, and physicochemical properties. In this study, we analyzed the physicochemical characteristics of honey produced by *A. florea* . Although extensive research has been conducted on the foraging behavior of *A. mellifera* in various regions of Iran, limited scientific literature addresses the physicochemical properties of honey produced by the dwarf honey bee, *A. florea* , within the country. Moreover, the bacterial composition of *A. florea* remains largely unexplored. The primary objective of this research was to utilize 16S rRNA gene sequencing to identify and characterize the bacterial species present in *A. florea* honey samples collected from diverse geographical regions of Iran. Additionally, this study aimed to examine the relationship between climatic factors and the physicochemical properties of honey, as well as to investigate the influence of climate on the diversity of probiotic bacteria present in honey.

## 2 Materials and Methods

**FIGURE 1:**
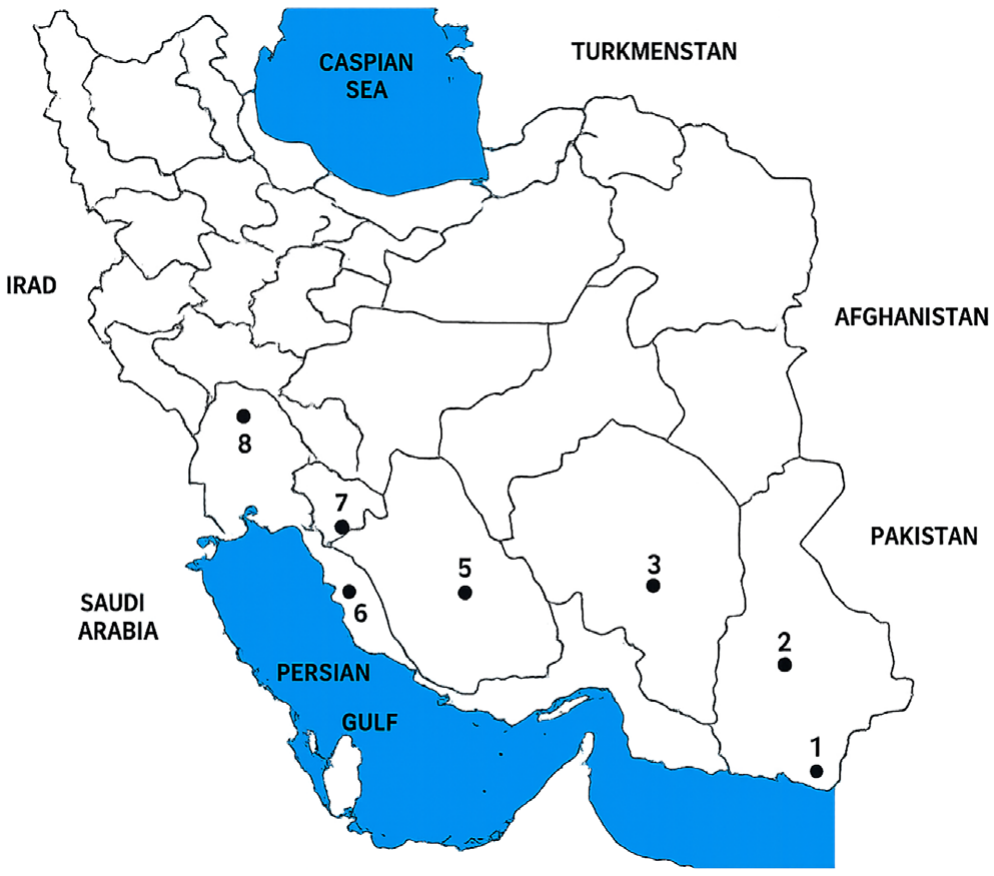
Sampling locations for *Apis florea* honey in Iran. The following locations are noteworthy within the region: 1. Chahbahar, 2. Iranshahr, 3. Jiroft, 4. Roudan, 5. Jahrom, 6. Boushehr, 7. Gachsaran, and 8. Dezfoul.

This study used honey derived from 40 colonies of dwarf honey bees sourced from 8 distinct regions of Iran (Figure 1) in the southern, southeastern, and southwestern areas. Sampling was conducted randomly in Boushehr, Chabahar, Dezful, Iranshahr, Jahrom, Jiroft, Gachsaran, and Rudan. From each colony, a one‐kilogram honey sample was aseptically obtained and transported to the laboratory at Razi Vaccine and Serum Research Institute in Karaj for analysis of physicochemical properties and the lactic acid bacteria content. In the regions of Boushehr, Dezful, and Jiroft, *Ziziphus* sp. were identified as the predominant floral source. In contrast, honey samples from Jahrom, Rudan, Iranshahr, Gachsaran, and Chabahar originated from a more diverse range of plants, including *Citrus*, *Astragalus*, *Prosopis*, *Eucalyptus*, and *Acacia* species.

In this study, honey samples produced in various regions of Iran were assessed for quality based on a range of physicochemical parameters. The analyses included measurement of acidity (Pascual‐Mate et al. 2018), pH (Pascual‐Mate et al. 2018), moisture content (Yegge et al. 2021), sucrose, fructose, glucose, proline, diastase activity, ash, and the presence of hydroxymethylfurfural (Feás et al. 2010).

### 2.1 Sucrose, Glucose, and Fructose Measurement

The difference in reducing sugar concentrations measured before and after hydrolysis was multiplied by a conversion factor of 0.95 to determine the sucrose content.

The glucose content was calculated by assessing the fructose‐to‐glucose ratio. Specifically, 20 mL of a 0.1 N iodine solution, 5 mL of 0.5 N NaHCO_3_, and 25 mL of the honey samples were combined and incubated in the dark for 15 min. Subsequently, 5 mL of 2 N H_2_SO_4_ was added, and the mixture was titrated with 0.1 N Na_2_S_2_O_3_. The glucose concentration (g) was determined based on the difference in the sodium thiosulfate consumption during titration.

Fructose content was calculated by subtracting the measured glucose content from the total reducing sugar content determined before hydrolysis (Hadhramie et al. 2023).

### 2.2 Proline Assay

Proline content was determined spectrophotometrically at 510 nm using ninhydrin, formic acid, and propanol as reagents (Sáez et al. 2004).

### 2.3 Determination of Diastase Enzymatic Activity in Honey Samples

Diastatic activity in honey reflects its enzymatic capacity, primarily attributed to diastase, which catalyzes the hydrolysis of starch into reducing sugars. This activity was measured using the Schade method. Briefly, a starch standard solution was added to each honey sample and incubated in a water bath at 40°C for 30 min. After incubation, approximately 1 mL of the reaction mixture was withdrawn and mixed with a few drops of iodine solution. The disappearance of the blue coloration indicated the hydrolysis of starch, confirming diastase activity. The decrease in blue color intensity was monitored spectrophotometrically at 660 nm. A time‐absorbance curve was generated, and a regression equation was used to determine the reaction time (*t*_*x*_, in minutes) required to reach an absorbance value of 0.235. The diastase number was then calculated using the formula Diastase Number = 300/*t*_*x*_ (Fallico et al. 2004).

### 2.4 Ash Content Measurement

Ash content was determined using the gravimetric method. Approximately 5 g of honey was accurately weighed into a crucible and incinerated in a muffle furnace at 550°C for 6 h, or until a constant weight was achieved to ensure complete combustion of organic matter. The remaining mineral residue was weighed, and the ash content was calculated as a percentage of the initial sample weight. This procedure was performed following the guidelines of the International Honey Commission (Bogdanov 2002).

### 2.5 Determination of Hydroxymethyl Furfural (HMF)

The hydroxymethylfurfural (HMF) content was determined using a spectrophotometric method. Samples were first treated with Carrez solution I (15 g of K_4_Fe (CN)_6_·3H_2_O dissolved in 100 mL distilled water) and Carrez solution II (30 g of Zn (CH_3_COO)_2_·2H_2_O dissolved in 100 mL distilled water). Subsequently, 0.2% sodium bisulfite was added to the control, while distilled water was added to the honey samples. Absorbance was measured at 284 and 336 nm against the control. HMF concentration was calculated by subtracting the absorbance at 336 nm from that at 284 nm (Ananias et al. 2013).

### 2.6 Culture Method

To isolate lactic acid bacteria (LAB), a 10% (w/v) honey solution was prepared in sterile normal saline and plated on MRS agar (de Man, Rogosa, and Sharpe; Oxoid) to promote LAB growth. The inoculated plates were incubated at 37°C for 3–4 days under anaerobic conditions using Anaerocult A gas packs in anaerobic jars (Merck, Darmstadt, Germany), according to the protocol of Tajabadi et al. (2011). To obtain pure cultures, 100 colonies exhibiting distinct morphological features were selected and further subcultured.

### 2.7 Biochemical Screening

Initial screening targeted the identification of LAB strains exhibiting Gram‐positive and catalase‐negative characteristics, by the criteria established by Coeuret et al. (2004). For long‐term preservation, selected isolates were stored at −18°C in MRS broth supplemented with 15% (v/v) glycerol, following the protocol described by Feizabadi et al. (2021) for subsequent analyses.

### 2.8 DNA Extraction

Genomic DNA was extracted using the QIA Gene kit protocol in combination with a modified method originally described by Ward (1994). DNA samples were dissolved in 150 μL of double‐distilled water and stored at −24°C. The quality of extracted DNA was evaluated by electrophoresis on a 1% agarose gel, while the DNA purity was determined via spectrophotometry by measuring the absorbance ratios at 260 and 280 nm.

### 2.9 PCR and Program

The 16S rDNA gene (approximately 1500 base pairs) was amplified using the 27F and 1492R primers (5’‐AGAGTTTGATCCTGGCTCAG‐3′ and 5’‐GGTTACCTTGTTACGACTT‐3′, respectively), which target the genus level of Lactobacilli Ward and Timmins 1999. Polymerase chain reaction (PCR) was carried out in a 20 μL reaction mixture, consisting of 0.1 μL Pfu DNA polymerase, 2 μL Pfu DNA polymerase buffer, 1.5 μL MgSO_4_, 0.25 μL of each primer, and 1 μL DNA template. The mixture was adjusted to the final volume with 14.9 μL deionized water, following the protocol described by Tajabadi et al. (2012) [Supporting Information]. PCR amplification was performed with an initial denaturation at 95°C for 3 min. This was followed by 40 cycles of denaturation at 95°C for 30 s, annealing at 55°C for 30 s, and extension at 72°C for 1 min. A final extension step was carried out at 72°C for 10 min, as outlined by Tajabadi et al. (2012).

The PCR products were separated on a 1% agarose gel and visualized with ethidium bromide staining. Amplified products were purified using the QIAquick PCR purification kit (QIAGEN, Hilden, Germany). Sequencing was performed using the 27F and 1492R primers by Macrogen (South Korea).

The resulting sequences, obtained from various isolates, were aligned and compared with 16S rRNA gene sequences in the GenBank database using the NCBI BLASTN tool (http://www.ncbi.nlm.nih.gov/BLAST/).

### 2.10 References Sequences Used in Phylogenetic Analyses

**FIGURE 2:**
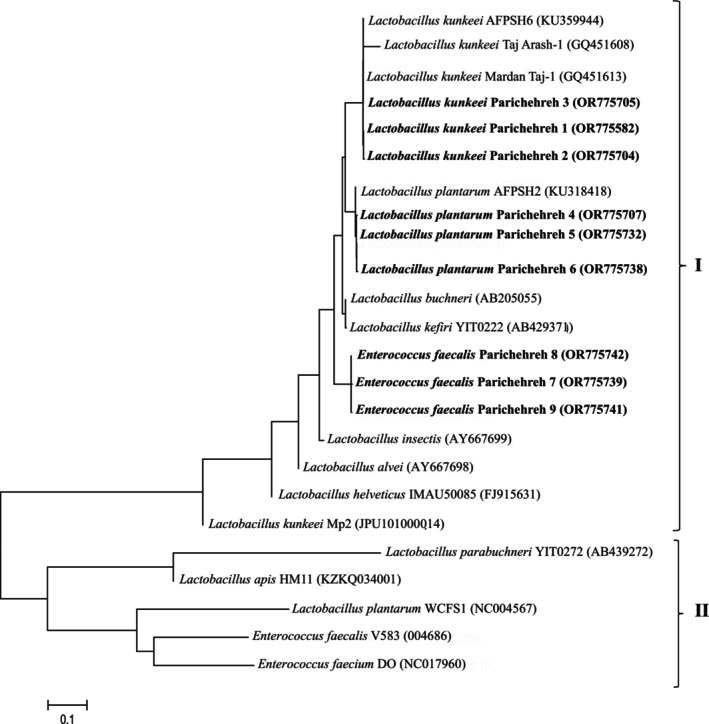
Phylogenetic analysis of lactic acid bacteria in honey samples collected from different regions of Iran. Phylogenetic tree based on a distance matrix analysis of 1275 positions in the 16S rDNA gene. The phylogenetic tree was constructed by ClustalW using the neighbor‐joining method within the MEGA (4) package (Tamura et al. 2007). Closely related type and reference strains are shown in parentheses together with accession numbers from GenBank. Bootstrap values based on 1000 resampling display the significance of the interior nodes and are shown at branch points. Cluster I *L. kunkeei, L. plantarum*, and *Enterococcus faecalis* group, and Cluster II as out group.

The following 16S rDNA gene sequences were used as outgroup in the phylogenetic analysis: *L. kunkeei* afpsh6 (KU359944), *L. kunkeei* Taj‐Arash 1 (GQ451608), *L. kunkeei* strain Mardan Taj‐1 (GQ451613), *L. plantarum* AFPSH2 (KU318418), *L. kefiri* YIT0222 (AB429371), *L. insectis* (AY667699), *L. alvi* (AY667698), *L. helveticus* (IMAU50085), *L. kunkeei* MP2 CONTIG006 (JPUI01000014) [Cluster I in Figure 2], *L. buchneri* CD034 (NC018610), *L. apis* HMA11 (KZKQ034001), *L. plantarum* WCFS1 (NC004567), *Enterococcus faecalis* V58 (004686), *Enterococcus faecium* DO (017960) [Cluster II in Figure 2] (Tajabadi et al. 2011; Vásquez et al. 2009).

### 2.11 Statistical Analyses

The Pearson correlation method was used to assess the relationship between climatic factors and honey characteristics. The Pearson correlation coefficient (*r*) is calculated using the following formula:

$$r=\frac{\text{COVxy}}{\mathrm{Sx}.\mathrm{Sy}}$$

where:

- *COV*_*xy*_ is the covariance between the two variables,
- *S*_*x*_ is the standard deviation of the first variable, and
- *S*_*y*_ is the standard deviation of the second variable.

A positive and statistically significant correlation coefficient indicates a direct relationship between the variables, whereas a negative and significant correlation coefficient reflects an inverse relationship. All physicochemical and microbiological measurements of honey samples were performed in triplicate. The meteorological data used in the analyses were obtained from the official websites of the respective provinces' meteorological centers.

Statistical evaluation of the data was carried out using one‐way analysis of variance (ANOVA), and differences between means were determined using Duncan's multiple range test at a 99% confidence level. Data analysis was performed using SPSS software, while graphical representations were created in Microsoft Excel 2013.

Phylogenetic analysis of the isolated LAB strains was performed using the maximum likelihood method, following the approach described by Sokal and Rohlf (1995). A neighbor‐joining phylogenetic tree was constructed in MEGA version 4 software (Tamura et al. 2007), with reliability assessed through 1000 bootstrap replicates.

## 3 Results and Discussion

The mean value for the physicochemical parameters of dwarf honeybee honey was as follows: acidity 15–18.5 (mEq/kg), pH 4.02–4.52, moisture content 14.23%–16.89%, sucrose 0%–3.8%, fructose 31%–36.89%, glucose 29.28%–39.19%, proline 208.48–347.27 (mg/kg), diastase activity 30–36.99 (°G), ash 0.09%–0.16%, and hydroxymethyl furfural (HMF) 0–19.46 (Table 1).

**TABLE 1:** The composition and physicochemical properties of honeys from different regions in Iran (mean ± SD).

Location	Acidity	pH	Moisture	Sucrose	Glucose	Fructose	Fru/Glc	Proline	Diastase	Ash	HMF
Boushehr	15 ± 1.96	4.52 ± 0.21	15.12 ± 2.42	0.00 ± 0.00	39.19 ± 2.13	35.81 ± 2.36	0.91 ± 0.006	286.06 ± 82.06	35.82 ± 2.15	0.09 ± 0.001	0.00 ± 0.00
Dezful	15.5 ± 2.12	4.47 ± 0.14	15.96 ± 2.75	2.01 ± 0.0085	29.28 ± 2.56	36.89 ± 3.65	1.26 ± 0.006	261.82 ± 22.1	36.24 ± 3.12	0.12 ± 0.002	13.47 ± 1.05
Iranshahr	18.5 ± 2.69	4.13 ± 0.17	15.98 ± 3.10	1.57 ± 0.0064	32.44 ± 2.36	31.85 ± 2.58	0.98 ± 0.0058	338.79 ± 79.58	36.73 ± 1.89	0.10 ± 0.001	2.25 ± 0.32
Rudan	14.5 ± 3.01	4.31 ± 0.41	16.89 ± 1.65	0.00 ± 0.00	35.14 ± 2.31	32.36 ± 3.21	0.92 ± 0.0062	208.48 ± 48.59	30.00 ± 2.56	0.09 ± 0.003	19.46 ± 1.52
Jiroft	17 ± 3.17	4.45 ± 0.33	15.72 ± 2.33	0.00 ± 0.00	30.63 ± 1.98	30.73 ± 1.98	1.00 ± 0.0075	256.97 ± 97.02	36.99 ± 3.03	0.16 ± 0.002	17.22 ± 2.12
Jahrom	15 ± 2.16	4.02 ± 0.65	14.23 ± 2.01	3.80 ± 0.0089	31.00 ± 2.76	32.00 ± 2.13	1.03 ± 0.0091	347.27 ± 27.72	36.99 ± 3.47	0.10 ± 0.002	3.43 ± 0.24
Chabahar	16.5 ± 2.15	4.13 ± 0.45	15.80 ± 3.25	1.40 ± 0.0035	31.00 ± 3.15	31.00 ± 3.31	1.00 ± 0.0063	332.00 ± 18.21	36.00 ± 1.98	0.09 ± 0.001	3.20 ± 0.25
Gachsaran	15.1 ± 2.72	4.05 ± 0.38	14.56 ± 1.94	3.60 ± 0.0092	31.00 ± 3.54	32.50 ± 3.01	1.05 ± 0.0092	345.00 ± 82.3	36.97 ± 2.03	0.15 ± 0.002	3.58 ± 0.52

The highest values for these parameters were generally observed in honey samples from Jiroft, Dezful, Rudan, Jahrom, and Boushehr, with Dezful exhibiting several of the maximum measurements. These regional differences in physicochemical characteristics are likely influenced by geographical factors, climate, and the diversity of local flora. Variations in temperature, humidity, and precipitation can affect both nectar composition and the microbial environment of honey production (Münstedt 2022). Furthermore, the abundance and diversity of nectar sources contribute to differences in sugar profiles, moisture content, and other key attributes. Collectively, these ecological factors underpin the observed regional variation in honey properties (Bayram 2019).

Minimum temperature was found to have a significant negative effect on sucrose (*p* < 0.01), proline (*p* < 0.05), and diastase (*p* < 0.05), but a significant positive effect on HMF (*p* < 0.01) (Table 2). Maximum temperature showed a significant positive association with HMF (*p* < 0.01), a negative (but not statistically significant) relationship with proline and diastase, and a positive, non‐significant association with sucrose (Table 2).

**TABLE 2:** Correlation between climate indices and honey traits.

Climate indices	T min	T max	Rh	Sunny days	Precipitation
Honey traits					
Sucrose	−0.586**	0.109	0.113	0.277	−0.340
Proline	−0.388*	−0.245	0.114	0.667**	−0.349*
Diastase	−0.477*	−0.268	0.018	0.356*	0.127
HMF	0.420*	0.614**	−0.049	−0.586**	0.591**

** Significant correlation at the 0.01 level.

* Significant correlation at the 0.05 level.

The temperature of the honey collection site can significantly influence sucrose, proline, and diastase activity (Alaerjani and Mohammed 2024). Elevated temperatures accelerate the conversion of sucrose to glucose and fructose through heat and enzymatic action, leading to reduced sucrose levels (da Silva et al. 2016). Additionally, the floral origin of the honey affects its initial sucrose content, as nectar sources vary in their natural sucrose concentrations (Liu et al. 2024; Pamminger et al. 2019) (Table 2).

Proline levels may decline at higher temperatures due to amino acid degradation (Manickavasagam et al. 2024). The proline content in honey is also influenced by the nectar source and regional environmental conditions, which determine the amino acid profile available to foraging bees (Ghosh et al. 2020; Parachnowitsch et al. 2019).

Higher temperatures can reduce diastatic activity by denaturing enzymes, whereas cooler conditions help preserve enzyme function (Bhure et al. 2025). However, the diastase activity additionally depends on floral sources and local environmental factors that shape bee foraging behavior (Sharma et al. 2023). Certain plants yield nectar with inherently higher amylase levels, contributing to regional variation in honey diastase activity (Kamal et al. 2019). Hydroxymethylfurfural forms in honey, especially when exposed to heat (Shapla et al. 2018). Elevated temperatures accelerate the Maillard reaction, resulting in higher HMF levels (Han et al. 2024). Honey harvested in warmer climates typically contains more HMF over time than honey from cooler regions (Godoy et al. 2022). The floral source and environmental conditions also shape the honey's initial sugar profile and acidity, which can further affect the rate of HMF formation (Fallico et al. 2004).

Relative humidity does not significantly affect any measured honey's properties. However, a greater number of sunny days was associated with higher proline content (*p* < 0.05) and diastase activity (*p* < 0.01). In contrast, sunny days correlated with lower HFM concentrations (*p* < 0.01). While sucrose levels appeared to increase with more sunny days, this effect was not statistically significant. Sunny days support the growth of flowering plants and stimulate nectar production by increasing photosynthesis (Phanindra et al. 2024). Consequently, honey collected during periods of abundant sunlight often contains higher sucrose levels due to the greater availability of nectar. Elevated temperatures and sunlight may also stress plants, leading to increased proline concentrations in nectar (Borghi et al. 2019). Additionally, the warmer conditions associated with sunny days can enhance diastase activity in honey (Lakhmili et al. 2024).

Precipitation significantly decreased sucrose (*p* < 0.05) and proline concentrations (*p* < 0.05), but increased HMF levels (*p* < 0.01). Rain reduces bee foraging and nectar availability, resulting in lower sucrose in honey (Lawson and Rands 2019; Vincze et al. 2024). Less environmental stress during rainy periods may also lead to reduced proline in nectar and, consequently, in honey (Torino 2022). Additionally, high precipitation and humidity can raise honey's moisture content and promote fermentation if the level exceeds 20%. Fermentation byproducts further influence HMF formation (Ananias et al. 2013).

Principal component analysis of climatic indicators revealed that precipitation, along with minimum and maximum temperatures, had the greatest influence on hydroxymethylfurfural (HMF) levels in honey. In contrast, the number of sunny days most strongly affected proline, sucrose, and diastase contents (Table 2).

Out of the lactic acid bacteria colonies cultured on MRS agar, 100 isolates were initially selected for preliminary biochemical characterization. From these, 34 representative isolates underwent 16S rRNA gene sequencing. The sequence alignment showed 99% similarity with reference strains in the NCBI database (Table 1). Phylogenetic analysis identified nine phenotypically distinct strains, classified into three species: *Lactobacillus kunkeei* , *L. plantarum* , and *Enterococcus faecalis* . These results indicate that these species dominate the lactic acid bacteria community in *A. florea* honey.

Within Cluster I (Figure 2), three unique phenotypes corresponded to *L. kunkeei* . Similarly, three distinct phylotypes matched *L. plantarum* in Cluster I based on sequence and cluster analysis (Table 3, Figure 2). Furthermore, three additional phylotypes were assigned as *E. faecalis*, also located in Cluster I (Figure 2 and Table 3). This distribution underscores the microbial diversity present in *Apis florea* honey.

**TABLE 3:** Identified bacterial phylotypes isolated from *Apis florea* honey samples.

Isolates	Identical number	Most closely related type strain	Sequence lengths and similarity	Accession numbers
Parichehreh 8	(4)	*Enterococcus faecalis* V583	(1200) 99%	OR775742
Parichehreh 9	(7)	*Enterococcus faecalis* V583	(1200) 99%	OR775741
Parichehreh 7	(2)	*Enterococcus faecalis* V583	(1200) 99%	OR775739
Parichehreh 6	(4)	*Lactobacillus plantarum* WCFS1	(1086) 98%	OR775738
Parichehreh 5	(8)	*Lactobacillus plantarum* WCFS1	(1086) 99%	OR775732
Parichehreh 4	(5)	*Lactobacillus plantarum* WCFS1	(1086) 99%	OR775707
Parichehreh 3	(7)	*Lactobacillus kunkeei* Mp2 contig 0016	(1095) 97%	OR775705
Parichehreh 2	(6)	*Lactobacillus kunkeei* Mp2 contig 0016	(1095) 99%	OR775704
Parichehreh 1	(4)	*Lactobacillus kunkeei* Mp2 contig 0016	(1095) 99%	OR77558

**FIGURE 3:**
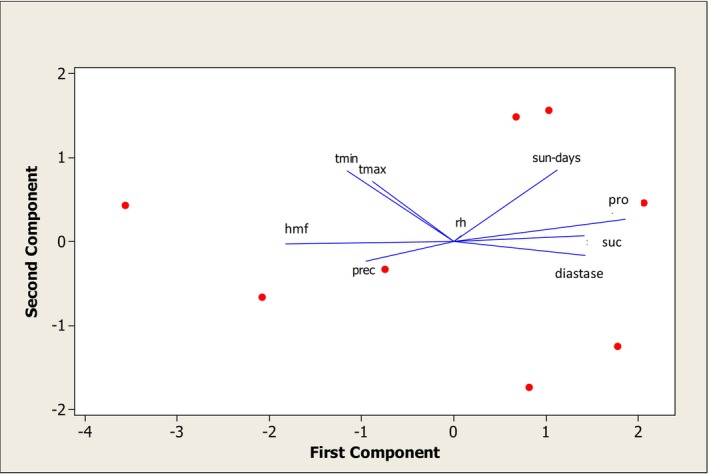
Examining the primary components influencing the relationship between climatic indicators and the abundance of physicochemical characteristics in honey samples.

The analysis identified *L. kunkeei* —a species commonly found in *Apis* honey—in multiple *A. florea* honey samples from Iranshahr, Chabahar, Jahrom, Rudan, Jiroft, Dezful, and Boushehr. *Lactobacillus plantarum* was detected in samples from Rudan, Jahrom, and Jiroft, with a notable presence in honey from citrus‐foraging areas. Additionally, *E. faecalis* was isolated from Gachsaran honey samples (Figure 2). The nucleotide sequences of the 16S rDNA gene from *Lactobacillus* and *Enterococcus* isolates have been submitted to the National Center for Biotechnology Information (NCBI) and are available under the accession numbers OR775742, OR775741, OR775739, OR775738, OR775732, OR775707, OR775705, OR775704, and OR775582 (Figure 3).

The diversity of microorganisms in honey is strongly influenced by bee pollen, nectar, and the bee gut (Endo et al. 2009; Anderson et al. 2013; Ambika Manirajan et al. 2016). Lactic acid‐producing microorganisms in honey primarily originate from these sources, particularly pollen, nectar, and the bee's gastrointestinal tract (Neveling et al. 2012). Several studies have examined the microbial composition of *A. mellifera* honey, focusing on both gut microbiota and the honey‐derived microorganism. For example, Mustar and Ibrahim (2022) identified 42 strains across six gener*a: Enterococcus*, *Micrococcus*, *Streptococcus*, *Pediococcus*, *Lactococcus*, and *Lactobacillus*. In a study of 25 *A. mellifera* honey samples, Seraglio et al. (2021) found that 25% of the isolates belonged to the genus *Lactobacillus*, with a distribution of *L. plantarum* (24%), *L. kazachii* (28%), and *L. acidophilus* (48%). The prevalence of *Lactobacillus* reported by Seraglio et al. (2021) exceeded that observed in our study. Additional isolates in their analysis included members of the genera *Bacillus*, *Enterococcus*, *Lactococcus*, and *Micrococcus*, as well as fungi and yeasts. Lashani et al. (2020) analyzed 88 honey samples and identified 27 strains of *L. kunkeei* , four strains of *L. plantarum* , two of *L. paracasei* , and single isolates of *L. brevis* , *L. rhamnosus* , *L. casei* , and *L. fermentum* . Using molecular techniques, Kwon et al. (2004) classified seven distinct LAB species— *L. acidophilus* , *L. delbrueckii* , *L. kazachii*, *L. gasseri* , *L. plantarum* , *L. reuteri* , and *L. rhamnosus* —with an accuracy of 93.6%. This study identified *L. kunkeei* as the dominant lactobacilli species, consistent with previous reports in various *Apis* species (Tajabadi et al. 2011, 2012; Vasquéz et al. 2012). In contrast, Feizabadi et al. (2021) found *L. plantarum* to be predominant in stored honey of *A. mellifera* . Vasquéz et al. (2012) recognized *L. kunkeei* as the primary species in the Apis genus (Vasquéz et al. 2012). Notably, *L. kunkeei* has been implicated in wine spoilage due to its inhibitory effects on yeast during fermentation (Mäki 2004).

Geographical variation in *Lactobacillus* isolation from honey—such as the recovery of *L. plantarum* from citrus orchard honey in Fars, Kerman, and Hormozgan provinces—likely reflects differences in regional climate, hydrology, and vegetation. Central and southern Iranian regions are characterized by dry, semi‐arid climates with desert and mountainous landscapes. Additionally, *L. pentosus* and *L. plantarum* contribute significantly to the fermentation of diverse plant, fish, and animal products (Curk et al. 1996; Zanoni et al. 1987), and show promise for both food industry applications and health benefits (Naidu et al. 1999).

In this study, a single phylotype from *A. florea* honey shared 99% genetic similarity with members of the *Lactobacillus* genus, suggesting a potentially novel strain. Three additional phylotypes associated with *Enterococcus* were also identified from *A. florea* honey, likely representing previously unrecognized strains. The presence of *L. kunkeei* , *L. plantarum* , and *E. faecalis* in *A. florea* honey is reported here for the first time.

These results provide foundational knowledge of lactic acid bacteria (LAB) in *A. florea* honey and broaden our understanding of its microbial characteristics. The identification of LAB with probiotic potential adds value to the known antimicrobial, antioxidant, and nutritional properties of this honey. These findings support the growing interest in functional foods, highlighting *A. florea* honey as a promising candidate for use in health‐focused food formulations or supplements aimed at promoting gut microbiota balance and digestive health.

Correlation analysis revealed a statistically significant but modest inverse relationship between temperature and the occurrence of *L. kunkeei* (*p* < 0.01). Although a negative correlation was also observed for *L. plantarum* and a positive trend for *E. faecalis* , these associations were not statistically significant (Table 4).

**TABLE 4:** Statistical relationship between climatic variables and the frequency of lactic acid bacteria in *Apis florea* honey.

Climate indices	T min	T max	Rh	Sunny days	Precipitation
Bacteria type					
*L. kunkeei*	−0.636**	−0.063	−0.143	0.303*	−0.135
*L. plantarum*	−0.174	0.262	−0.281	−0.151	−0.029
*E. faecalis*	0.191	0.303*	−0.047	−0.609**	0.692**

** Significant correlation at the 0.01 level.

* Significant correlation at the 0.05 level.

Maximum temperature showed a positive and statistically significant association with the abundance of *E. faecalis* (*p* < 0.05). This variable also positively influenced *L. plantarum* , but this effect was not statistically significant. Relative humidity displayed a negative relationship with all three bacterial species; however, these associations were not significant.

The frequency of sunny days had a positive and statistically significant effect on *L. kunkeei* abundance (*p* < 0.05), but a negative and statistically significant impact on *E. faecalis* (*p* < 0.01). A decrease in *L. plantarum* abundance was also observed with more sunny days, though this did not reach significance. Precipitation was positively and significantly associated with *E. faecalis* abundance (*p* < 0.01), but negatively affected *L. kunkeei* and *L. plantarum* ; these latter effects were not statistically significant (Table 4).

**FIGURE 4:**
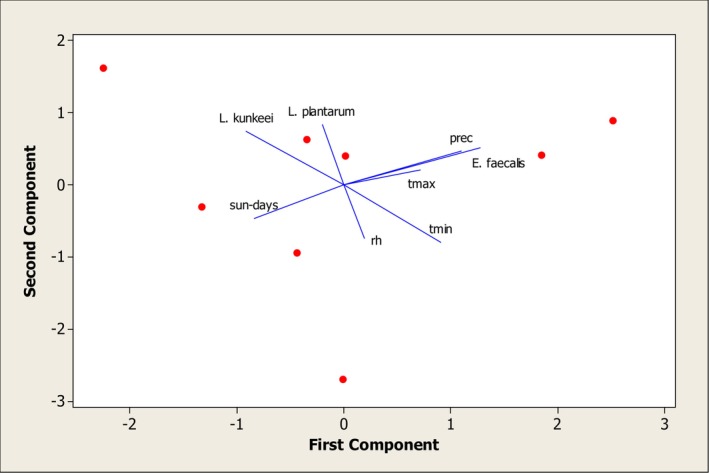
Principal component analysis of the influence of climatic indicators on the abundance of lactic acid bacteria in honey samples.

Principal component analysis (PCA) was used to assess the combined effects of climatic variables on bacterial populations (Figure 4). The results showed that maximum temperature and precipitation substantially increased *E. faecalis* abundance. In contrast, the number of sunny days had the greatest positive effect on *L. kunkeei* , while *L. plantarum* was least affected by climatic conditions.

## 4 Conclusion

This study offers comprehensive insights into the physicochemical and microbiological characteristics of *Apis florea* honey, underscoring its potential as a functional food with health‐promoting properties. Regional variations in climate, specifically temperature, precipitation, and sunlight exposure, significantly affected key quality indicators, including proline content, diastase activity, and hydroxymethylfurfural (HMF) levels, which are essential for assessing honey's freshness, nutritional value, and safety. Moreover, the isolation and molecular identification of probiotic‐associated lactic acid bacteria such as *Lactobacillus kunkeei* , *L. plantarum* , and *Enterococcus faecalis* are particularly significant. These microorganisms not only enhance the microbial diversity of *A. florea* honey but also suggest potential gastrointestinal health benefits, supporting its application in functional foods and dietary supplements. Overall, these findings deepen our understanding of how environmental factors influence honey composition and highlight the promise of *A. florea* honey as a natural, bioactive ingredient in health‐oriented food systems.

## Author Contributions

**Shabnam Parichehreh:** conceptualization (lead), funding acquisition (lead), methodology (equal), writing – original draft (equal). **Gholamhosein Tahmasbi:** data curation (lead), supervision (lead). **Maryam Asnaashari:** formal analysis (equal), investigation (equal), software (equal), validation (equal), visualization (equal). **Fatemeh Ahmadi:** formal analysis (equal), investigation (equal), software (equal), validation (equal), visualization (equal). **Mohammad Eslampanah:** formal analysis (equal), investigation (equal), software (equal), validation (equal), visualization (equal).

## Ethics Statement

This study does not have any human or animal testing.

## Conflicts of Interest

The authors declare no conflicts of interest.

## Supporting information


**Table S1.** PCR ingredients, volumes, and concentration for each PCR tube.

## Data Availability

The data that support the findings of this study are available from the corresponding author upon reasonable request.
