# Supplementary material for: Climatic Determinants of Physicochemical Traits and Probiotic Composition in Dwarf Honeybee ( Apis florea ) Honey
Source: Food Sci Nutr. 2025 Jul 16;13(7):e70640. doi: 10.1002/fsn3.70640 (PMC12264384; doi:10.1002/fsn3.70640)
Supplement: Supplementary file 1 — Table S1. PCR ingredients, volumes, and concentration for each PCR tube. [file FSN3-13-e70640-s001.docx]

| Supplementary material: Table I: PCR ingredients, volumes, and concentration for each PCR tube | | |
| --- | --- | --- |
| Component | Volume (µl) | Standard |
| Pfu DNA polymerase | 0.1 | 1 X |
| Pfu DNA polymerase buffer | 2 | 10 X |
| MgSO_4_ | 1.5 | 1.5 mM |
| Forward primer | 0.25 | 0.25 mM |
| Reverse primer | 0.25 | 0.25 mM |
| DNA template | 1 | 0.4 ng |
| Sterile Distilled water | 14.9 | - |
| Total | 20.00 | - |
